# A coherent quantum annealer with Rydberg atoms

**DOI:** 10.1038/ncomms15813

**Published:** 2017-06-22

**Authors:** A. W. Glaetzle, R. M. W. van Bijnen, P. Zoller, W. Lechner

**Affiliations:** 1 Institute for Theoretical Physics, University of Innsbruck, Innsbruck A-6020, Austria; 2 Institute for Quantum Optics and Quantum Information of the Austrian Academy of Sciences, Innsbruck A-6020, Austria; 3 Centre for Quantum Technologies, National University of Singapore, 3 Science Drive 2, Singapore 117543, Singapore; 4 Clarendon Laboratory, University of Oxford, Parks Road, Oxford OX1 3PU, UK

## Abstract

There is a significant ongoing effort in realizing quantum annealing with different physical platforms. The challenge is to achieve a fully programmable quantum device featuring coherent adiabatic quantum dynamics. Here we show that combining the well-developed quantum simulation toolbox for Rydberg atoms with the recently proposed Lechner–Hauke–Zoller (LHZ) architecture allows one to build a prototype for a coherent adiabatic quantum computer with all-to-all Ising interactions and, therefore, a platform for quantum annealing. In LHZ an infinite-range spin-glass is mapped onto the low energy subspace of a spin-1/2 lattice gauge model with quasi-local four-body parity constraints. This spin model can be emulated in a natural way with Rubidium and Caesium atoms in a bipartite optical lattice involving laser-dressed Rydberg–Rydberg interactions, which are several orders of magnitude larger than the relevant decoherence rates. This makes the exploration of coherent quantum enhanced optimization protocols accessible with state-of-the-art atomic physics experiments.

## Abstract (web summary)

It is an open challenge to implement a coherent quantum annealer with fully programmable all-to-all connectivity. Here the authors present a scheme based on enhanced Rydberg-dressed interactions between neutral atoms in an optical lattice which is programmable by individual addressing of local fields.

Quantum annealing is a quantum computing paradigm with the aim to solve generic optimization problems^1^,^2^,^3^,^4^, where the cost function corresponds to the energy of an infinite-range Ising spin glass^5^. Finding the optimal solution of the problem is thus equivalent to determining the ground state of the spin glass. In quantum annealing, this task is accomplished by adiabatic passage of a system of *N* spins in the instantaneous ground state of a Hamiltonian (denoted logical spin model) of the form


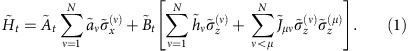


Here 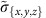 are Pauli spin operators, and time-dependent scheduling functions 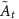 and 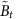 deform 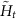 from a trivial initial Hamiltonian with 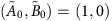 and transverse local fields 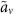, into the spin glass Hamiltonian with 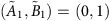, where the optimization problem is encoded in local fields 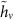 and infinite–range interactions 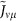
5.

Current quantum annealing implementations^6^ focus on the adiabatic preparation of classical states with thermally assisted adiabatic quantum protocols. Here, we propose a setup for coherent annealing with the aim to open an experimental route to adiabatic and non-adiabatic protocols. For example, non-adiabatic and fully coherent sweeps allow for counter-diabatic protocols^7^ with the potential to improve scaling laws in quantum annealing considerably, as well as the recently introduced hybrid annealing method^8^. While thermally assisted annealing is suitable to prepare classical ground states or mixtures, a fully coherent annealer can also be used to prepare quantum superpositions from Hamiltonians with degenerate ground states^9^. A coherent protocol with non-stoquastic^10^ driver terms opens a promising route to highly efficient protocols for optimization problems^11^.

A major challenge in implementing equation (1) is posed by the required individually programmable long–range interactions 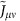, which is in contradiction to polynomially decaying interactions in cold atoms and molecule setups. Adopting the LHZ architecture^12^, the infinite-range spin glass is translated to a lattice spin model, where new physical spins 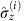 represent the relative orientation of two logical spins 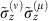 of equation (1). If two logical spins are aligned in parallel, that is, 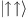 or 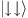, then the corresponding physical spin is in state 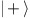, while if the logical spins are aligned anti-parallel, that is, 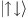 or 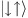, then the physical spin is in state 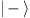. The major advantage of this approach is that the interaction energy of a pair of logical spins can now be implemented with a local field acting on a single physical spin.

A general optimization problem in the LHZ architecture becomes


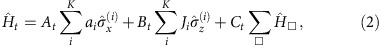


with new schedule functions *A*_*t*_, *B*_*t*_ and *C*_*t*_, and transverse fields *a*_*i*_. Physical spins are arranged on a square lattice (see blue spheres in Fig. 1), where the index *i* labels the entries of the matrix 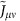. The number of physical spins *K* equals the number of connections in the original model, which is quadratically larger than in the original problem, for example, *K*=*N*(*N*−1)/2 for all-to-all connected graphs. This enlarged state space contains states, where collections of physical spins encode conflicting relative orientations of the logical spins. These states can be locally identified and energetically penalized by four-body constraints *H*_□_ at each plaquette □ of the square lattice, such that at the end of the sweep plaquettes either contain all an even^12^, or all an odd^13^ number of spins in the 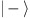 state, thus realising an even or odd parity representation of equation (2). This ensures that the final ground state of the LHZ Hamiltonian (2) corresponds to the final ground state of the logical Hamiltonian (1), and thus to the optimal solution of the optimization problem. Importantly, the optimization problem is now encoded in local fields 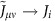, corresponding to single-particle energy shifts. We show that the above model for a programmable quantum annealer can be emulated in an atomic Rydberg setup, which builds on the remarkable recent advances towards realizing complex spin models with cold atoms in lattices interacting via designed Rydberg–Rydberg interactions^14^,^15^,^16^,^17^,^18^.

## Results

### Four-body parity constraints

The key challenge of implementing 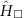 is resolved with Rydberg atoms by combining the odd parity representation^13^ of equation (2) with enhanced Rydberg–Rydberg dressing^19^ schemes in a two-species mixture^20^,^21^. In the odd parity representation, the sum of the four spins at each plaquette is either 2 or −2. We introduce a single ancilla qubit 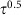 at each plaquette, which can compensate the two associated energies and make odd parity plaquette states degenerate ground states of the constraint Hamiltonian 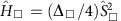, with stabilizer operators 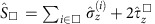, and energy gap 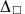. This allows to implement the four-body gauge constraints via appropriately designed two-body Ising interactions between physical and ancilla qubits.

Here we consider a more general and robust form of 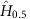, consisting of all combinations of two-body interactions along the edges and diagonals of the plaquette, as well as with the ancilla spin (see Fig. 2(a)), of the form


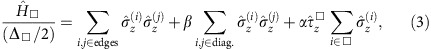


where *α* and *β* are relative interaction strengths compared to spin interactions along the plaquette edge. The energy spectrum *E*_□_ of a single plaquette Hamiltonian is shown in Fig. 2(b), as a function of the parameters *α* and *β*. Importantly, there exists a parameter regime 0<2−*β*<*α*<2+*β* with 0<*β*<1, where the odd parity states are degenerate and have a lower energy than the even parity states. As the precise value of the gap in Fig. 2 is not relevant, as long as it exceeds all other energy scales, 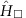 is quite robust against small variations in interaction strengths, and the parameters *α*, *β* need not be fine-tuned.

### Interaction design

**Figure 1:**
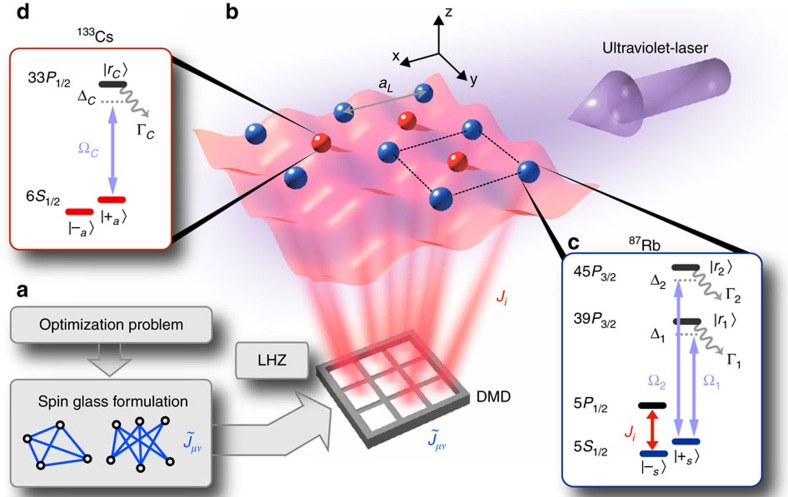
Quantum Annealing with Rydberg atoms. (**a**) The cost function of a general optimization problem in the form of a spin glass with infinite-range interactions 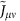 is encoded in the Lechner–Hauke–Zoller (LHZ) architecture in local fields *J*_*i*_. (**b**) Rubidium (blue) and Caesium (red) atoms are trapped in a square lattice geometry representing physical and ancilla spins, respectively, where the spin degree of freedom is encoded in two long-lived hyperfine states 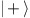 and 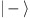, illustrated in insets (**c**,**d**). The programmable local fields *J*_*i*_ are induced by AC stark shifts from laser coupling the 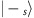 state to low-lying 5*P* states using a digital mirror device (DMD). The four-body gauge constraints at each plaquette (for example, the black dotted square) are engineered using off-resonant laser coupling of the 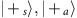 states to Rydberg *P*-states 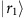, 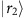 or 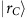 and require only uniform illumination of the system with ultraviolet laser light. Γ_1_, Γ_2_ and Γ_C_ are the decay rates from the excited states.

**Figure 2:**
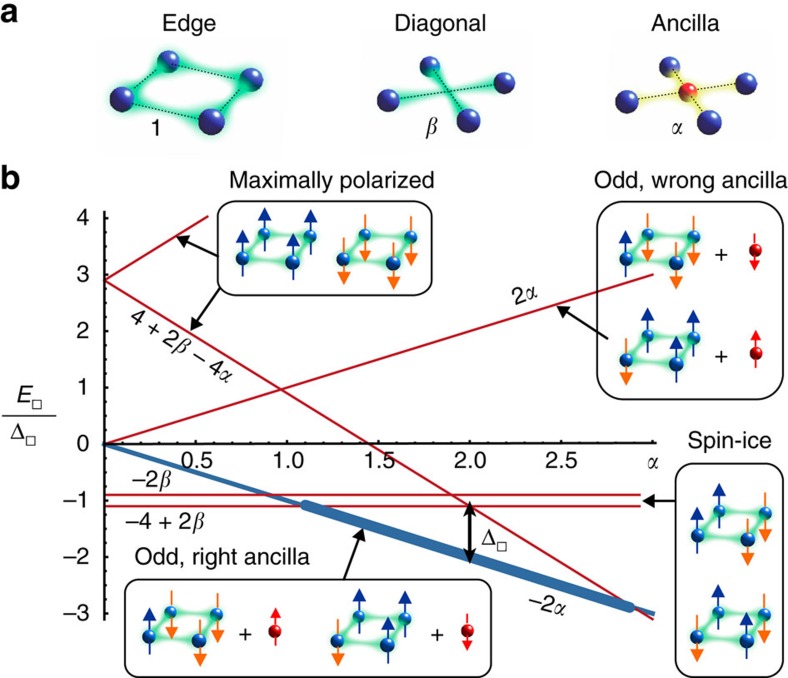
Parity plaquette constraints. (**a**) Four-body interactions between physical spins (blue) of the same plaquette are constructed from two-body interactions between physical spins of strength 1 along the edge of the plaquette (left), interactions of strength *β* along the diagonal (middle) and additional interactions of strength *α* between an ancilla qubit (red) located at the centre of each plaquette and the surrounding physical qubits (right). (**b**) Eigenenergies *E*_□_ of the Hamiltonian of equation (3), as a function of the physical spin-ancilla interaction strength *α* for a particular 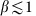. Odd parity eigenstates with the right (lower left inset) or wrong (upper right inset) ancilla orientation have an energy ±2*α*. The maximally polarized states with all four physical spins up or down (upper left inset) have energy 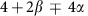, while the spin-ice states are independent of the ancilla interaction *α* and have constant energies −2*β* and −4+2*β*. The thick blue line indicates the window of interest where the odd parity states are the ground states of the plaquette Hamiltonian.

In the Rydberg quantum annealer illustrated in Fig. 1, qubits are encoded in two long-lived hyperfine ground states 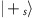, 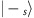 of ^87^Rb and 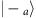, 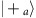 of ^133^Cs, representing physical and ancilla spins, respectively. These states are trapped in a deep optical or magnetic square lattice with unity filling and frozen motion^22^,^23^. Consecutive loading schemes of rubidium and caesium have been successfully demonstrated^21^, which could be combined with the recent remarkable progress in trapping atoms in almost arbitrary 2D geometries using optical tweezers^22^,^23^. Alternatively, it was suggested^20^ that rubidium and caesium atoms are trapped in the same optical lattice created by counter-propagating laser beams with wavelength *λ*_L_≈820 nm. This particular wavelength is blue detuned for Cs atoms, which will be trapped at the intensity minima, but red detuned for Rb atoms, which will be trapped at the intensity maxima. Thus the atoms are trapped in an alternating pattern, as illustrated in Fig. 1.

In particular, we choose the 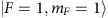 and 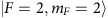 hyperfine states of the 5^2^*S*_1/2_ ground state manifold of ^87^Rb and the 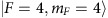 and 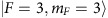 hyperfine states of the 6^2^*S*_1/2_ ground state manifold of ^133^Cs. The first term of equation (2) can be realized with a coherent driving field of amplitude *a*_*i*_ coupling the 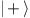 and 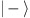 states for both physical and ancilla atoms, written in a rotating frame. The second term is obtained using single-particle AC-Stark shifts from off-resonant laser coupling of the 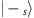 spin state to low-lying 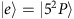 states using a digital micro-mirror device^24^. The strenght of these fields can be easily varied as a function of time, thus implementing the sweep coefficients *A*_*t*_ and *B*_*t*_ in equation (2).

To implement the two-body interactions of equation (3) we turn to the technique of Rydberg dressing^25^,^26^,^27^,^28^, where off-resonant laser light weakly admixes some Rydberg character into the ground state levels 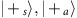, leading to an effective interaction between them. For large laser detunings, the Rydberg dressing acts as a perturbation and the dressed levels predominantly retain their ground state character and remain trapped^29^. Interactions between two spins *i* and *j* arise, as spatially dependent light shifts 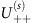 and 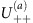 of the dressed pair states 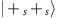 and 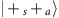, respectively. These pair states are coupled via two photon excitations to doubly excited Rydberg states. Because of multipole–multipole interactions, the energies of the doubly excited Rydberg states vary strongly as a function of the relative position *R*_*ij*_ of the atoms, and can exceed typical laser detunings and coupling strengths even at micrometre distances. This strongly affects the light shifts of the dressed pair ground states, thus endowing them with an effective interaction.

**Figure 3:**
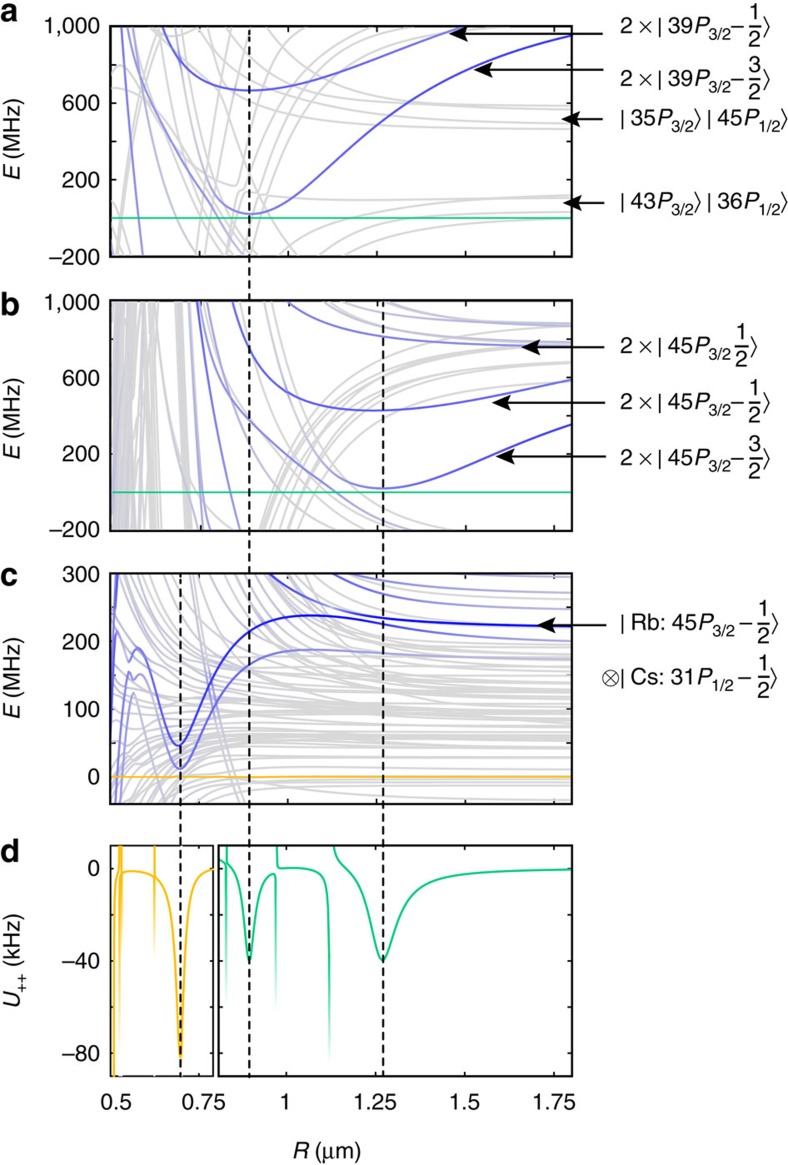
Enhanced Rydberg dressing. Rydberg-Rydberg interaction energies *E* around the (**a**) 
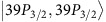
 and (**b**) 
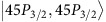
 states of ^87^Rb and **c** the mixed 
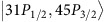
 Rydberg state of ^133^Cs and ^87^Rb, in a magnetic field of *B*_*z*_=26 G along the *z* axis. The intensity of the blue colouring indicates the overlap with the laser-targeted Rydberg states. The most strongly coupled pair potentials feature distinct local minima, which can be exploited in an enhanced Rydberg dressing scheme by detuning the Rydberg dressing laser such that, in the rotating frame, the pair ground state energies (green line for the 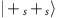 state in **a**,**b**, and yellow line for the 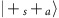 state in (**c**) approach the minima of the potential wells. This configuration leads to relatively large and strongly peaked level shifts of the dressed ground states, as can be seen in **d** which shows the resulting interaction potential *U*_++_ between two Rydberg-dressed Rb–Cs (yellow) and Rb–Rb (green) ground state atoms. Drastically enhanced peaked-like interactions appear at 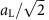, *a*_L_ and 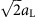, thereby realizing the required plaquette interaction of equation (3) for *α*=2 and *β*=1. Vertical dashed lines connect the extrema in the ground state level shifts to the corresponding Rydberg potential minima in the excited state manifold. Various resonances due to accidental degeneracies of the ground states with weakly coupled excited states are visible as well, but these are of negligible width and occur away from lattice sites.

Here, we propose to couple the 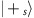 and 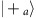 states of Rb and Cs using single photon transitions to the *m*_*J*_=−1/2 magnetic sublevels of Rydberg *P* states^16^,^17^,^30^,^31^,^32^,^33^,^34^, with laser light that is linearly polarized along the *z* axis and thus retains the symmetry of the lattice geometry. Figure 3 shows the Rydberg pair energies in the presence of multipole interactions, in the vicinity of (a) the 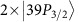 and (b) the 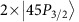 Rydberg states of ^87^Rb and (c) the mixed 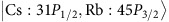 Rydberg states. The pair potentials are obtained from an exact diagonalization calculation (Supplementary Note 2), including interactions up to quadrupole–quadrupole and dipole–octupole couplings. In the regime of strong interactions, the Rydberg states are mixed by the multipole couplings, and each energy level corresponds to a superposition of Rydberg states with a variety of quantum numbers. The blue colouring in Fig. 3 represents the overlap with the laser targeted *m*_*J*_=−1/2 states, and is therefore indicative of the effective coupling strength to the pair ground states. Although there are many energy levels in the regime of interest, we see that at distances >0.5 μm only a handful of potential curves is significantly coupled by the dressing laser, while the vast majority of states (grey curves) is only coupled with negligible strengths.

The particular Rydberg *P* pair states shown in Fig. 3(a–c) feature pronounced potential wells which enable an enhanced Rydberg dressing scheme^19^. For this purpose, we tune the (two-photon) detuning of the dressing laser such that, in the rotating frame, the energy of the ground pair states (green line in Fig. 3(a,b) for 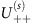, and yellow line in panel (c)) is close to a potential minimum. This gives rise to pronounced light shifts of the pair ground states at the point of closest approach, as shown in Fig. 3(d), while at the same time producing negligible light shifts elsewhere. The particular Rydberg states in Fig. 3(a–c) are chosen such that the dressed ground state potentials 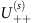 and 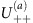 plotted in Fig. 3(d) show peaks at distances 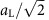, *a*_L_ and 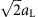, commensurate with a square lattice geometry with *a*_L_=0.89 μm. Accidental crossings of the doubly excited Rydberg levels with the pair ground state energy give rise to resonances in the dressed state level shifts, but these either occur at distances which are not present in the lattice geometry, or they are of negligible width on behalf of a vanishing laser coupling strength to the crossing states.

### Rydberg annealer

The final spin–spin interactions, following from the light shifts described in the previous Section, are given by


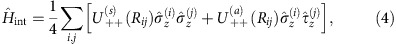


apart from additional single-particle corrections to the local fields. The height of the two peaks of 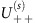 at *a*_L_ and 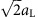 for Rb-Rb (green line in Fig. 3(d)) and of 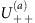 at 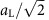 for Rb–Cs (yellow line in Fig. 3(d)) can be tuned by varying the Rabi frequencies and detunings of the dressing laser. In particular, we choose Rabi frequencies Ω_1_=Ω_2_=2*π* × 35 MHz, Ω_*C*_=2*π* × 30 MHz and detunings Δ_1_=−2*π* × 618 MHz, Δ_2_=−2*π* × 373 MHz and Δ_*C*_=2*π* × 175 MHz which leads to light-shifts of 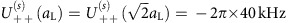 for Rb–Rb and 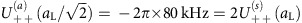 for Rb–Cs (Supplementary Note 2). We note that an external magnetic field and small vertical offset of the Cs atoms are used to obtain the final interaction strengths and precisely align the potential extrema with plaquette distances (Supplementary Note 2).

All interactions between atoms (spins and ancillas) that are not part of a common plaquette are at least two orders of magnitude smaller than the interactions within a plaquette. This allows us to restrict the sum in equation (4) to pairs of atoms belonging to the same plaquette, thus realizing 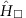 of equation (3) for the optimal parameters *α*=2 and *β*=1. For the above system parameters, we obtain a final energy gap Δ_□_=−2*π* × 20 kHz. Note that the energy gap is negative, which can be easily accounted for by a sign change of all local fields and making the annealer adiabatically follow the maximum energy state, instead of the minimum.

Because of the finite lifetime of the Rydberg states, the dressed ground state interactions come at a cost of an effective decoherence rate 1/*τ*_0_ for each qubit. However, as there is only a small Rydberg component admixed, the effective decay rate is also only a correspondingly small fraction of the bare Rydberg decay rate. Ultimately, the figure of merit for fully coherent operation of the quantum annealer is the ratio of the attained interaction strength versus the effective decay rate. In the enhanced dressing scheme this ratio becomes particularly favourable and is of the order of 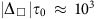 for the system parameters above (Supplementary Note 2).

**Figure 4:**
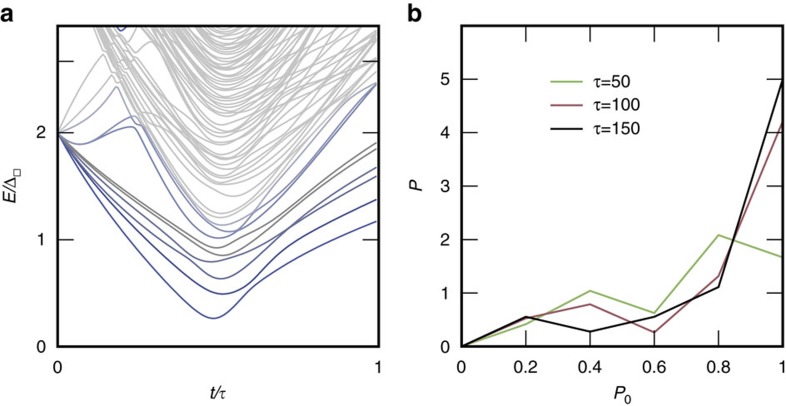
Spectrum and success probabilities. (**a**) Illustration of the time-dependent spectrum for the minimal instance shown in Fig. 1. (**b**) Histogram of the success probability, that is, the probability *P*_0_ to populate the ground state at final time *τ* for different sweep times.

Using the above potentials we demonstrate numerically the feasibility of the Rydberg annealer for the minimal instance (Fig. 1) with eight qubits and three ancillas. Figure 4 depicts the time dependent spectrum in reduced units for an instance of Hamiltonian equation (2) for random 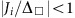. The sweep functions *A*_*t*_, *B*_*t*_ and *C*_*t*_ are simple linear functions. Note, that the efficiency can be considerably increased by adopting non-linear sweep functions. In Fig. 4 all energies are given relative to the ground state energy. The pronounced minimal gap is an order of magnitude smaller than the gap in the final state. Figure 4(b) shows the histogram of the success probability 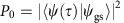, defined as the overlap of the final state 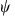 with the ground state 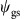, averaged over *N*_*r*_=40 random instances for different sweep times far below the decoherence times *τ*<*τ*_0_/*K*. For the fastest switching time 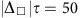 the average success probability is 75% and approaches unity for slower sweeps.

## Discussion

The proposed implementation of a quantum annealer with ultracold Rydberg atoms in optical lattices provides a platform for adiabatic quantum computing, featuring a highly controllable environment to explore the many-body adiabatic passage, the role of entanglement and effects of decoherence during the annealing sweep. The large lifetimes of Rydberg dressed atoms enable coherent quantum annealing as an alternative to the current paradigm of quantum enhanced thermal annealing^35^. We anticipate that due to the coherent evolution the number of spins in future experiments can readily be extended well beyond the minimal example presented here, by using shorter annealing cycles with many repetitions^36^, or by employing counter-diabatic driving schemes that could greatly increase the attained fidelities^7^. Finally, our proposal allows to realize atomic quantum simulators of arbitrary infinite-range Ising spin glass models (see, for example, references in ref. 37), and the combination of multi-color Rydberg-dressed interactions with two-species mixtures has applications in realizing 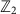 lattice gauge theories beyond the present example^38^.

### Data availability

Data that support the findings of this study are available from the authors on reasonable request.

## Additional information

**How to cite this article:** Glaetzle, A. W. *et al*. A coherent quantum annealer with Rydberg atoms. *Nat. Commun.* **8**, 15813 doi: 10.1038/ncomms15813 (2017).

**Publisher’s note:** Springer Nature remains neutral with regard to jurisdictional claims in published maps and institutional affiliations.

## Supplementary Material

Supplementary Information Supplementary Figures, Supplementary Notes and Supplementary References.
