# Supplementary material for: A coherent quantum annealer with Rydberg atoms
Source: Nat Commun. 2017 Jun 22;8:15813. doi: 10.1038/ncomms15813 (PMC5489683; doi:10.1038/ncomms15813)
Supplement: Supplementary Information — Supplementary Figures, Supplementary Notes and Supplementary References. [file ncomms15813-s1.pdf]

I Supplementary note 1

A general quantum annealing problem with an infinite-range spin glass Hamiltonian consisting of N logical spins $\tilde{\sigma}$ with K connections has the form

$$\tilde{H}_t^{(\log)} = \tilde{A}_t \sum_{\nu=1}^{N} \tilde{a}_\nu \tilde{\sigma}_x^{(\nu)} + \tilde{B}_t \sum_{\nu<\mu}^{N} \tilde{J}_{\mu\nu} \tilde{\sigma}_z^{(\nu)} \tilde{\sigma}_z^{(\mu)} \tag{1}$$

with scheduling functions $\tilde{A}_t$ and $\tilde{B}_t$, local transverse fields $\tilde{a}_\nu$ and programmable infinite-range interactions $\tilde{J}_{\mu\nu}$.

Using the LHZ architecture it can be mapped on a spin model

$$\hat{H}_t^{(\mathrm{LHZ})} = A_t \sum_{i}^{K} a_i \hat{\sigma}_x^{(i)} + B_t \sum_{i}^{K} J_i' \hat{\sigma}_z^{(i)} + C_t \sum_{\square} \Delta_\square \prod_{i\in\square} \hat{\sigma}_z^{(i)}, \tag{2}$$

with K physical spins $\hat{\sigma}$, arranged on a square lattice (green circles in Supplementary Fig. 1), and problem independent 4-body interactions between spins belonging to the same plaquette $\square$ of the square lattice (red dots in Supplementary Fig. 1). Here, a_i are transverse local fields and $\Delta_\square$ is the four-body interaction strength (which, for simplicity, we assume to be equal for all spins and plaquettes, respectively), and C_t is the scheduling function of the constraints. In the LHZ architecture the programmable interaction matrix $\tilde{J}_{\mu\nu}$ is translated to programmable single-particle energy shifts J_i' which correspond to the entries of the matrix $\tilde{J}_{\mu\nu}$.

In an odd parity representation the 4-body interactions are resolved by introducing an ancilla qubit $\hat{\tau}^\square$ in the middle of each plaquette with fine-tuned 2-body interactions of the form

$$\hat{H}_t^{(\mathrm{odd})} = A_t \left(\sum_{i}^{K} a_i \hat{\sigma}_x^{(i)} + \sum_{\square} a_\square \hat{\tau}_x^\square \right) + B_t \sum_{i}^{K} J_i \hat{\sigma}_z^{(i)} + C_t \sum_{\square} \Delta_\square \left(\sum_{i\in\square} \hat{\sigma}_z^{(i)} + 2\hat{\tau}_z^\square \right)^2, \tag{3}$$

where the local fields J_i are the entries of the matrix $(-1)^{\mu(\nu-\mu)} \tilde{J}_{\mu\nu}$, and $a_\square$ is a transverse local field driving the ancilla spins.

In the following we illustrate the annealing sweep and the time-dependent spectrum of Supplementary Eq. (3) for the minimal instance of 8 logical qubits (Rubidium atoms) and 3 ancilla qubits (Cesium atoms) which makes a total of 11 qubits arranged on three plaquettes illustrated in Fig. 1 of the main text and Supplementary Fig. 1. This setup corresponds to 4 all-to-all connected logical qubits in Supplementary Eq. (1).

The order of the indices of the local fields is from bottom to top (e.g. $\tilde{J}_{12} \to J_3$). In the

minimal instance depicted in Supplementary Fig. 1 these are the three plaquettes formed by physical qubits $(1,3,4,6)$, $(2,4,5,7)$ and $(6,4,7,8)$. In the odd parity scheme, the phase factor $(-1)^{\mu(\nu-\mu)}$ will flip the sign of the fourth local field, i.e. $J_4' = -J_4$, such that the parity of all plaquettes is odd.

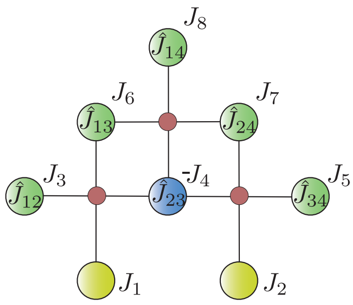

Supplementary Figure 1: Numerical example: The minimal instance shown in Fig. 1 in the main text corresponds to a fully connected spin glass with $N = 4$ spins. In the LHZ architecture, the entries of the interaction matrix $\tilde{J}_{\mu\nu}$ in Supplementary Eq. (1) translate to local fields J_i. Here, the index i label local fields ordered from bottom to top. The bottom row of local fields are fixed to large positive numbers $J_1 = J_2 = 5|\Delta_\Box|$. The minimal instance discussed here consists of $N = 8$ spins with 3 ancilla spins accounting for the three 4-body plaquette constraints (red dots).

We demonstrate the feasibility of quantum annealing for this small instance numerically calculating the success probability P_0 for constant $\Delta_\Box$ and a_i. This is the overlap of the wave function at final time T with the ground state wavefunction $\psi_{\rm gs}$

$$P_0 = |\langle\psi(T)|\,\psi_{\rm gs}\rangle|^2. \quad (4)$$

The statistics of P_0 is obtained by solving the time-dependent Schrödinger equation with Hamiltonian of Supplementary Eq. (3), using the Rydberg interaction potentials given in the main text and in Sec. II of the supplemental information, with exact diagonalization and explicitly including the dynamics of the ancilla qubits. Statistics is taken from $N_i = 40$ random instances of an interaction matrix with $J_i/|\Delta_\Box| \in [-0.5, 0.5]$. The schedule functions $A_t = T - t$ and $B_t = C_t = t$ both interpolate linearly from 0 to T. Note, that this linear ramp is a pessimistic toy model and more sophisticated choices of the schedule function could considerably increase the success probability. The bottom two qubits $i = 1$ and $i = 2$ fixing the gauge constraints in the parity architecture are explicitly part of the dynamics and subject to strong local field $J_1 = J_2 = 5|\Delta_\Box|$. The total time is $|\Delta_\Box|T = 50, 100, 150$, which is significantly shorter than the expected single particle coherence time of the Rydberg states of Rubidium and Cesium which

is $|\Delta_\Box|\tau_0 \approx 10^3$. The results shown in Fig. 3 in the main text show that for this system size the overlap with the ground state wave function is between 70% and 90% for the given parameters.

II Supplementary note 2

II.A Rydberg-Rydberg potentials

In this section, we outline the calculation of the Rydberg-Rydberg interaction potentials that underly the effective interactions described in the main text. Throughout the discussion we set $\hbar = 1$ for notational clarity.

The excited Rydberg states $|e\rangle = |n(\ell s)jm_j\rangle$ of the valence electron with spin $s = 1/2$ of alkali atoms, such as rubidium or cesium, are well described by their principal quantum number n, orbital angular momentum ℓ, and total angular momentum j with its projection m_j along the quantization axis. The states $|e\rangle$ are eigenstates of a single particle Hamiltonian

$$\hat{H}_A = \sum_e \left(E_e^{(0)} + \Delta E_{m_j}\right) |e\rangle\langle e|. \tag{5}$$

The bare atomic energies $E_e^{(0)} = -E_{\mathrm{Ryd}}/(n-\delta_{\ell,j})^2$ are different for rubidium and cesium atoms, and are determined by their respective quantum defects $\delta_{\ell,j}$, see e.g. Refs. [1, 2] and [3], respectively. We include level shifts $\Delta E_{m_j} = \mu_B g_j B_z m_j$ due to a magnetic field $\mathbf{B} = B_z \mathbf{z}$ which lifts the Zeeman degeneracy and sets the quantization axis along the z-axis. Here, $\mu_B = 1.4\ h$ MHz/G is the Bohr magneton and g_j is the Lande factor for the Rydberg level.

The Hamiltonian describing the internal-state dynamics of two particles separated by a spatial vector $\mathbf{R} = (R, \vartheta, \varphi)$, to good approximation, given by

$$\hat{H} = \hat{H}_A^{(1)} \otimes \hat{I} + \hat{I} \otimes \hat{H}_A^{(2)} + \hat{V}_{\mathrm{mm}}(\mathbf{R}). \tag{6}$$

with the first term of each operator product acting on the first particle, and the second term acting on the second. The single particle atomic Hamiltonians $\hat{H}_A^{(1)}$ and $\hat{H}_A^{(2)}$ can describe rubidium or cesium atoms. The third term corresponds to multipolar interactions coupling the internal states of the two particles. We write the matrix elements of $\hat{V}_{\mathrm{mm}}$ in a basis of pair

states, using the shorthand notation $|e_a e_b\rangle = |l_a j_a m_{j,a}\rangle \otimes |l_b j_b m_{j,b}\rangle$, and obtain

$$
\begin{aligned}
\langle 12|\,\hat{V}_{\rm mm}(\mathbf{R})\,|34\rangle = & \sum_{k_1,k_2=1}^{\infty} (-1)^{k_2}\mathcal{R}_{1,3}(k_1)\mathcal{R}_{2,4}(k_2)\sqrt{\frac{4\pi(2k_1+2k_2)!}{(2k_1)!(2k_2)!(2k_1+2k_2+1)}} \\
& \times (-1)^{l_{tot}-2s+k_1+k_2+m_{j,1}+m_{j,2}}\sqrt{\hat{l}_1\hat{l}_2\hat{l}_3\hat{l}_4\hat{j}_1\hat{j}_2\hat{j}_3\hat{j}_4} \\
& \times \sum_{p_1=-k_1}^{k_1}\sum_{p_2=-k_2}^{k_2} C^{k_1+k_2,p_1+p_2}_{k_1 p_1,k_2 p_2} Y_{k_1+k_2,p_1+p_2}(\hat{R}) \\
& \times \begin{pmatrix} l_1 & l_3 & k_1 \\ 0 & 0 & 0 \end{pmatrix} \begin{pmatrix} j_3 & k_1 & j_1 \\ m_{j,3} & p_1 & -m_{j,1} \end{pmatrix} \begin{Bmatrix} j_3 & k_1 & j_1 \\ l_1 & s & l_3 \end{Bmatrix} \\
& \times \begin{pmatrix} l_2 & l_4 & k_2 \\ 0 & 0 & 0 \end{pmatrix} \begin{pmatrix} j_4 & k_2 & j_2 \\ m_{j,4} & p_2 & -m_{j,2} \end{pmatrix} \begin{Bmatrix} j_4 & k_2 & j_2 \\ l_2 & s & l_4 \end{Bmatrix}, \qquad (7)
\end{aligned}
$$

where we have defined $l_{tot} = \sum_{i=1}^{4} l_i$, and $\hat{l}_i = 2l_i + 1$, as well as $\hat{j}_i = 2j_i + 1$. With C and Y we denote Clebsch-Gordan coefficients and spherical harmonics, respectively, and

$$\mathcal{R}_{a,b}(k) = \int dr r^2 \psi_a^*(r) r^k \psi_b(r) \qquad (8)$$

is the radial matrix element of k-th multipole moment with $\psi_{a,b}(r)$ the radial wavefunctions of the Rydberg states $|e_a\rangle$ and $|e_b\rangle$. The terms $k_1 = k_2 = 1$ in the sum of (7) correspond to the familiar dipole-dipole interactions, whereas $k_1, k_2 = 2, 3$ correspond to quadrupole and octupole interaction terms. For the interatomic distances in this work, the dipole-dipole terms are dominant, although the Rb-Cs mixed potentials required also terms up to quadrupole-quadrupole and dipole-octupole terms to be taken into consideration.

We proceed by selecting a large basis set of pair states $|e_a e_b\rangle = |l_a j_a m_{j,a}\rangle \otimes |l_b j_b m_{j,b}\rangle$, which are product states of eigenstates of the single particle Hamiltonians $\hat{H}_A^{(1)}$ and $\hat{H}_A^{(2)}$, with energies E_{ab} given by the sum of the corresponding single-atom energies. In this basis, the Hamiltonian (6) turns into a large but sparse matrix. We diagonalize $\hat{H}$ numerically for a range of distances R and fixed orientation angle ϑ. The basis set is chosen sufficiently large, containing $\sim 10^4$ states, to ensure convergence of eigenstates and eigenvalues down to distances of $R \sim 0.5\mu$m. The diagonalization procedure yields distance dependent molecular eigenenergies $E_\mu(R)$ depending only on the radial distance R, which are the interaction potentials plotted in Fig. 3(a) in the

main text. Simultaneously the corresponding molecular eigenstates $|\mu(\mathbf{R})\rangle$ are computed,

$$|\mu(\mathbf{R})\rangle = \sum_{ab} c_{ab}^{(\mu)}(\mathbf{R})\, |e_a e_b\rangle\,, \tag{9}$$

which are superpositions of pair product states $|e_a e_b\rangle$ with coefficients $c_{ab}^{(\mu)}(\mathbf{R})$ depending on (R, ϑ, φ). The coloring of the curves in Fig. 3(a) of the main text is indicative of the overlap with the laser targeted Rydberg state, $|\langle e_{\lambda 1} e_{\lambda 2}|\mu(\mathbf{R})\rangle|^2 = |c_{\lambda 1,\lambda 2}^{(\mu)}(\mathbf{R})|^2$, with $\lambda 1, \lambda 2 = \{$'1', '2', 'C'$\}$ defining the particular Rydberg states [see e.g. Fig. 1 in the main text].

II.B Rydberg dressing potentials

Having obtained the molecular eigenstates $|\mu(\mathbf{R})\rangle$ and their energies $E_\mu(R)$, we can now proceed to calculate the light shifts of the ground state levels resulting from laser coupling to the excited state manifold.

The laser couplings are characterized by a Rabi frequency, Ω_λ, and detuning from a targeted Rydberg level, Δ_λ. The subscript $\lambda = \{$'1', '2', 'C'$\}$ indexes the three distinct laser couplings discussed in the main text:

λ = '1' pertains to the laser coupling the $|{+}\rangle = |F = 2, m_F = -2\rangle$ hyperfine ground state of Rb to the $|e_1\rangle = \left|39P_{3/2}, m_J = -1/2\right\rangle$ Rydberg state,

λ = '2' indexes the laser coupling of the $|{+}\rangle$ state to the $|e_2\rangle = \left|45P_{3/2}, m_J = -1/2\right\rangle$ Rydberg state,

λ = 'C' refers to the laser coupling of the $|{+}_a\rangle = |F = 4, m_F = -4\rangle$ hyperfine ground state of an ancilla Cs atom to the $|e_C\rangle = \left|31P_{1/2}, m_J = -1/2\right\rangle$ Rydberg state.

All lasers propagate in the xy-plane, with linear polarization along the z-axis coinciding with the quantization axis and magnetic field direction. This geometry is chosen such that the total system and resulting interaction potentials are rotationally symmetric along the z-axis, and in particular the energies of the plaquette configurations are invariant under rotation and mirroring operations.

In the Rydberg dressing limit, the laser coupling is far off-resonant with $\Omega_\lambda \ll |\Delta_\lambda|$, such that

the effect of the laser coupling is perturbative with an associated small parameter $\epsilon = \Omega_\lambda/|\Delta_\lambda|$. In the following we only consider the laser-coupled ground states $|{+_s}\rangle, |{+_a}\rangle$, as the uncoupled ground states $|{-_s}\rangle, |{-_a}\rangle$ play no role in the interaction calculation. To describe the state of two laser coupled ground state particles, we work in a basis consisting of pair states $|g_1g_2\rangle$, where both particles are in one of the two ground state $|{+_s}\rangle$ or $|{+_a}\rangle$, i.e. $g_1, g_2 = +_s, +_a$. This basis is extended with pair states $|g_1e_{\lambda2}\rangle, |e_{\lambda1}g_2\rangle$ where one of the two particles is in the ground state while the other is excited to the laser-targeted Rydberg state, with $\lambda1, \lambda2 = \{$'1', '2', 'C'$\}$. Due to appropriately chosen laser frequency and polarization we only couple to these targeted Rydberg states. Finally, the basis also contains the molecular states $|\mu(\mathbf{R})\rangle$ we obtained numerically in the previous section.

The ground pair state $|g_1g_2\rangle$ is defined to have an energy 0. The atomic Rydberg states of particle 1 are defined in a rotating frame corresponding to the laser frequency $\omega_{\lambda1}$, such that the near-resonant, laser-targeted Rydberg state $|e_{\lambda1}\rangle$ has an energy $-\Delta_{\lambda1}$. Similarly, the Rydberg states of particle 2 have their energy defined relative to the energy $-\Delta_{\lambda2}$ of the $|e_{\lambda2}\rangle$ state in the rotating frame of the laser transition $\lambda2$. The molecular Rydberg states $|\mu(\mathbf{R})\rangle$ therefore have an energy

$$\delta^{(\mu)}(R) = E_\mu(R) - E_{\lambda1\lambda2} - \Delta_{\lambda1} - \Delta_{\lambda2}. \tag{10}$$

Expressed in the basis described above, the two-particle Hamiltonian thus becomes

$$\hat{H} = -\Delta_{\lambda1}|e_{\lambda1}g_2\rangle\langle e_{\lambda1}g_2| - \Delta_{\lambda2}|g_1e_{\lambda2}\rangle\langle g_1e_{\lambda2}| + \sum_\mu \delta^{(\mu)}(R)|\mu(\mathbf{R})\rangle\langle\mu(\mathbf{R})| + \hat{H}_L(\mathbf{R}), \tag{11}$$

where the operator $\hat{H}_L$ is the laser coupling

$$\begin{aligned}\hat{H}_L(\mathbf{R}) = \frac{\Omega_{\lambda1}}{2}|e_{\lambda1}g_2\rangle\langle g_1g_2| + \frac{\Omega_{\lambda2}}{2}|g_1e_{\lambda2}\rangle\langle g_1g_2| + \text{h.c.}\\ + \sum_\mu\left[\frac{\Omega^{(\mu)}_{\lambda1}(\mathbf{R})}{2}|\mu(\mathbf{R})\rangle\langle g_1e_{\lambda2}| + \frac{\Omega^{(\mu)}_{\lambda2}(\mathbf{R})}{2}|\mu(\mathbf{R})\rangle\langle e_{\lambda1}g_2|\right] + \text{h.c.},\end{aligned}$$

where the first line contains terms coupling the pair ground state $|g_1g_2\rangle$ to the single excited pair states, and the second line couples the singly excited states to the molecular states. The effective coupling strength to the molecular states,

$$\Omega^{(\mu)}_\lambda(\mathbf{R}) = \Omega_\lambda c^{(\mu)}_{\lambda1,\lambda2}(\mathbf{R}), \tag{12}$$

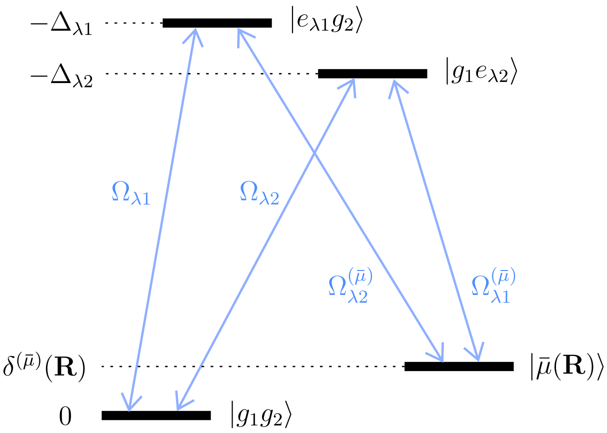

Supplementary Figure 2: Atomic level scheme: Energy levels involved to obtain the dressed ground state potentials. The double ground state $|g_1, g_2\rangle$ is laser-coupled to the $|e_{\lambda 1}, g_2\rangle$ or $|g_1, e_{\lambda 2}\rangle$ single-excited Rydberg state with Rabi frequencies $\Omega_{\lambda 1}$ and $\Omega_{\lambda 2}$ and energies $\Delta_{\lambda 1}$ and $\Delta_{\lambda 2}$, respectively. These state can be further excited to doubly-excited Rydberg states $|\bar{\mu}(\mathbf{R})$ with position dependent Rabi frequencies $\Omega^{(\bar{\mu})}_{\lambda 1}$ and $\Omega^{(\bar{\mu})}_{\lambda 2}$. Close to a molecular potential well the energy $\delta^{(\bar{\mu})}(R)$ can be much smaller than the single particle detunings Δ_λ resulting in an enhanced light shift of the dressed $|g_1, g_2\rangle$ state.

with $\lambda = \lambda 1, \lambda 2$, is reduced with a factor $c^{(\mu)}_{\lambda 1, \lambda 2} \leq 1$, and is additionally dependent on the distance.

The light shifts of the pair ground state presented in the main text, Fig. 3(d), are calculated by numerically diagonalizing Hamiltonian (11). Here, however, it is instructive to analyze the light shifts perturbatively. In particular, we consider the case where there is one dominant molecular state, denoted $\mu = \bar{\mu}$, with significant effective laser coupling strength and lying close to 0 in energy. This is precisely the situation described in the main text in the vicinity of the point of closest approach of the molecular wells to the pair ground state, as depicted in Fig 3(a)-(c) in the main text. Supplementary Fig. 2 illustrates the relevant pair states, their energies and the couplings between them. Note that in the perturbative limit, higher order processes involving three or more particles are negligible.

To second order in the laser coupling, the light shift of the pair ground state is constant and independent of distance,

$$E^{(2)} = \frac{1}{2}\Omega_{\lambda 1}\epsilon_{\lambda 1} + \frac{1}{2}\Omega_{\lambda 2}\epsilon_{\lambda 2}, \tag{13}$$

where we have defined $\epsilon_\lambda = \Omega_\lambda / 2\Delta_\lambda$. Thus far, the laser light shift is merely a single particle effect. Interactions enter in fourth order perturbation theory, when we consider processes involving couplings to the molecular state $|\bar{\mu}(\mathbf{R})\rangle$. The resulting contribution to the light shift is

(ignoring terms contributing to the single particle light shift)

$$E_{\text{int}}^{(4)} = \epsilon_{\bar{\mu}}^2 \delta^{(\bar{\mu})}(R), \tag{14}$$

where we have defined

$$\epsilon_{\bar{\mu}} = \frac{\Omega_{\lambda 1}\Omega_{\lambda 2}^{(\bar{\mu})}}{4\delta^{(\bar{\mu})}(R)} \left(\frac{1}{\Delta_{\lambda 1}} + \frac{1}{\Delta_{\lambda 2}} \right) = \frac{\Omega_{\lambda 2}\Omega_{\lambda 1}^{(\bar{\mu})}}{4\delta^{(\bar{\mu})}(R)} \left(\frac{1}{\Delta_{\lambda 1}} + \frac{1}{\Delta_{\lambda 2}} \right). \tag{15}$$

Clearly, choosing the laser detunings such that $\delta^{(\bar{\mu})}(R)$ becomes small, boosts the interaction strength. The above perturbative expression is valid as long as $\delta^{(\bar{\mu})}(R) \gg \epsilon_\lambda \Omega_\lambda^{(\bar{\mu})}$. A particular situation where the perturbative treatment breaks down occurs when a molecular state crosses the zero energy level. At such a point, pairs of Rydberg atoms are resonantly excited by the laser, instead of the intended weak admixture. The system parameters in the main text are chosen such that this situation is avoided, by ensuring that no significant resonances occur at lattice distances. We included checking the 'cross' potentials, i.e. the molecular potentials for the case $\lambda 1 =$'1', and $\lambda 2 =$'2'.

A final quantity of interest is the Rydberg state admixing into the atomic ground states, as this determines the dominant decoherence rate in the system. Again, the state admixing can be calculated in the pair basis and perturbative limit discussed above. The new dressed pair ground state, denoted $|\widetilde{g_1 g_2}\rangle$, becomes

$$|\widetilde{g_1 g_2}\rangle = |g_1 g_2\rangle + \epsilon_{\lambda 1} |e_{\lambda 1} g_2\rangle + \epsilon_{\lambda 2} |g_1 e_{\lambda 2}\rangle - \epsilon_{\bar{\mu}} |\bar{\mu}(\mathbf{R})\rangle + \mathcal{O}\left(\frac{\Omega_{\lambda 1,\lambda 2}^2}{\Delta_{\lambda 1,\lambda 2}^2} \right), \tag{16}$$

where we have ignored the normalization, and used $\delta^{(\bar{\mu})}(R) \ll \Delta_{\lambda 1,\lambda 2}$ to truncate the expansion after the third term. Assuming for simplicity a single decoherence rate Γ for all Rydberg states, we see that the second and third term in Supplementary Eq. (16) each introduce an effective decoherence rate $\epsilon_\lambda^2 \Gamma$ to the dressed pair state, whereas the third term introduces a decoherence rate $2\epsilon_{\bar{\mu}}^2 \Gamma$, where the factor 2 in front stems from the fact that the state $|\bar{\mu}(\mathbf{R})\rangle$ has two particles in the excited state. The total decoherence per particle is evidently

$$\Gamma_{\text{eff}} = \frac{1}{2}(\epsilon_{\lambda 1}^2 + \epsilon_{\lambda 2}^2 + 2\epsilon_{\bar{\mu}}^2)\Gamma. \tag{17}$$

The figure of merit for realizing fully coherent operation of the quantum annealer, i.e. the

ratio of interaction strength versus decoherence rate (per particle), is now readily computed from the results obtained above. Using Supplementary Eqs. (14) and (17), we have that

$$\frac{E_{\rm int}^{(4)}}{\Gamma_{\rm eff}} = \frac{2\epsilon_{\bar{\mu}}^2 \delta^{(\bar{\mu})}(R)}{(\epsilon_{\lambda 1}^2 + \epsilon_{\lambda 2}^2 + 2\epsilon_{\bar{\mu}}^2)\Gamma}. \tag{18}$$

For the system parameters employed in the main text we thus obtain:

$\lambda 1 = \lambda 2 = 1$: dressing to $|e_1\rangle = |39P_{3/2}, m_J = -1/2\rangle$, for which the single particle lifetime is $\tau_1 = 1/\Gamma_1 = 54\mu$s [4], and at the minimum of the selected potential well $\delta^{(\bar{\mu})}(R) = 2.5$MHz and $c^{(\bar{\mu})} \simeq 0.32$, leading to a final figure of merit $\frac{E_{\rm int}^{(4)}}{\Gamma_{\rm eff}} \simeq 8.0 \times 10^2$,"

$\lambda 1 = \lambda 2 = 2$: dressing to $|e_2\rangle = |45P_{3/2}, m_J = -1/2\rangle$, with single particle lifetime $\tau_2 = 1/\Gamma_2 = 75\mu$s [4], and $\delta^{(\bar{\mu})}(R) = 5.5$MHz and $c^{(\bar{\mu})} \simeq 0.28$, leading to a final figure of merit $\frac{E_{\rm int}^{(4)}}{\Gamma_{\rm eff}} \simeq 1.9 \times 10^3$,

$\lambda 1 = 2, \lambda 2 = C$: simultaneous dressing of Rb to $|e_2\rangle = |45P_{3/2}, m_J = -1/2\rangle$ and Cs to $|e_C\rangle = |31P_{1/2}, m_J = -1/2\rangle$, with averaged single particle lifetime $\tau_C = 1/\Gamma_C = 48\mu$s [4], and $\delta^{(\bar{\mu})}(R) = 4.5$MHz and $\epsilon_{\bar{\mu}}^2 \simeq 0.08$, leading to a final figure of merit $\frac{E_{\rm int}^{(4)}}{\Gamma_{\rm eff}} \simeq 1.1 \times 10^3$.

A final point of attention concerns a small vertical offset of the Cs atoms in the z-direction, which is necessary for getting an exact match of the potential peaks with the lattice geometry with lattice spacing $a_L = 0.89\mu$m. The Rb-Cs potential for the chosen parameters has its peak at 0.69μm, which is slightly larger than the required $a_L/\sqrt{2} = 0.63\mu$m. A vertical offset of the Cs atoms of ≈ 280nm compared to the plane of the Rb atoms would compensate for this difference.

Supplementary References

[1] C. J. Lorenzen and K. Niemax, Precise quantum defects of nS, nP and nD Levels in Cs I, Zeitschrift für Physik A Atoms and Nuclei **315**, 127 (1984).

[2] W. Li, I. Mourachko, M. W. Noel, and T. F. Gallagher, Millimeter-wave spectroscopy of cold Rb Rydberg atoms in a magneto-optical trap: Quantum defects of the ns, np, and nd series, Phys. Rev. A **67**, 052502 (2003).

[3] K.-H. Weber and C. J. Sansonetti, Accurate energies of nS, nP, nD, nF, and nG levels of neutral cesium, Phys. Rev. A **35**, 4650 (1987).

[4] I. I. Beterov, I. I. Ryabtsev, D. B. Tretyakov, and V. M. Entin, Quasiclassical calculations of blackbody-radiation-induced depopulation rates and effective lifetimes of Rydberg nS, nP, and nD alkali-metal atoms with n≤80, Phys. Rev. A **79**, 052504 (2009).
